# Personalized risk-adapted models in prostate cancer during active surveillance using MRI—a narrative review

**DOI:** 10.1007/s00330-025-11518-z

**Published:** 2025-04-04

**Authors:** Davide Maffei, Caroline M. Moore

**Affiliations:** 1 Division of Surgery & Interventional Science, University College London, London, UK; 2 Department of Urology, University College London Hospitals NHS Foundation Trust, London, UK

**Keywords:** Prostatic neoplasms, Active surveillance, Watchful waiting

## Abstract

**Abstract:**

The presence of histologically defined prostate cancer (PCa) is common and rises with age. Nevertheless, histological evidence of PCa does not always lead to clinically evident or life-threatening disease, and we know that PSA-based population screening can find twice the prevalence of PCa than would present via clinical routes. The PROTECT study randomized men diagnosed through PSA screening, to surgery, radiotherapy, or active monitoring. At 15 years, PCa-related deaths ranged from 2.1% in the surgery group to 2.9% in the active monitoring group, while the risk of death from other causes was 22%. Modern PCa diagnosis uses MRI to determine who needs a biopsy and how it is done.

Multiparametric MRI can selectively detect PCa of higher grade and volume, which is more likely to be associated with progression, metastases, and death, and hence to benefit from treatment. MRI and MRI-targeted biopsies are recommended prior to enrolment in active surveillance (AS) programs for accurate risk classification. The UCLH AS cohort based on per-cause MRI evaluations has shown baseline Gleason grade and MRI index lesion visibility to be strong predictors of progression to treatment. Serial evaluation with MRI has been codified with the PRECISE recommendations to support MRI-based monitoring during AS.

AS recommendations have extended to certain favorable-intermediate risk cancers. The Movember International Consensus Meeting has determined a dynamic risk-stratified AS approach to be the highest-ranked research priority.

We review the impact of MRI on AS from patient selection to risk stratification, and the development of MRI-led personalized AS protocols.

**Key Points:**

***Question*** *Standardized protocols are not able to address the heterogeneity of men on active surveillance for PCa and are burdened by unnecessary examinations*.

***Findings*** *MRI can improve the selection of men for active surveillance, predict time to treatment, and risk-stratify patients at risk of progression*.

***Clinical relevance*** *MRI-led risk-adapted protocols may reduce the burden of active surveillance on patients, improve adherence, and reduce healthcare costs*.

## Introduction

The diagnosis, risk stratification, and management of prostate cancer (PCa) have changed significantly over time.

A paper discussing new approaches to PCa diagnosis from Memorial Hospital in 1933 described risk stratification using the volume of residual urine, duration of symptoms and pain, or demonstrable metastases [1]. They reported the use of needle aspiration of the prostate for the cytological definition of PCa.

The next development was the use of Gleason grading in 1967 [2]. Gleason described the architectural appearance of PCa from benign to the most malignant and this approach has continued, with minor modifications, since then [3]. Whilst this is the bedrock of PCa diagnosis and risk stratification, recently some have questioned whether Gleason Grade 3 + 3 should be called cancer or defined in an alternate way because of its extremely low potential for metastasis and PCa-related death [4].

The introduction of prostate-specific antigen (PSA) testing in the 1990s further changed the detection of PCa, as there was now a way to find men with localized PCa before symptom onset [5, 6]. While this has significantly reduced the number of men presenting with metastatic disease it has led to a huge rise in the diagnosis of cancer where the need for treatment is, at best, uncertain.

Albertsen described the natural history of untreated PCa and demonstrated elegantly that at the lower Gleason grades, particularly in older men, the risk of dying of other causes was much higher than the risk of dying of PCa [7]. This finding was confirmed in the PROTECT study in which men diagnosed with PCa through a PSA screening study were randomized to surgery, radiotherapy, or active monitoring, irrespective of risk group [8]. Active monitoring was intentionally much less intense than current active surveillance, which typically includes routine biopsy at timed intervals. It included a repeat 6-core transrectal ultrasound (TRUS) biopsy if there was a 50% increase in PSA. The diagnosis was based on a standard TRUS biopsy, which is likely to have underestimated the grade and burden of disease compared to current magnetic resonance imaging (MRI)-based diagnostic practices, and analysis of those in the radical prostatectomy arm showed that 20.3% were D’Amico intermediate and 8.1% high-risk disease.

At 15 years, PCa-related deaths ranged from 2.1% in the surgery group to 2.9% in the active monitoring groups. The risk of death from other causes was 22%. Men were estimated to be eight to ten times more likely to die of other causes than PCa in the first 15 years.

In this article, we summarize the evidence on the use of MRI to guide risk-stratified follow-up schedules in active surveillance for PCa.

## What is clinically significant PCa?

Defining the boundary between significant and insignificant PCa has been and remains a significant challenge. The first definition of clinically significant PCa came from Stamey’s work in 1983 where he saw that 8% of men in the SEER database had PCa. He then analyzed the whole mount prostate histopathology from a series of 139 consecutive cystoprostatectomies [9], considered to be representative of PCa in the general SEER population, and found that 55 (40%) of these prostates harbored PCa. He concluded that the top 8% of these cancers would be the ones that would present as clinically significant, which corresponded to a clinically significant volume of 0.5 cc on whole mount analysis.

In 1994 Epstein and colleagues analyzed 156 radical prostatectomies for T1c disease, (detected not on digital rectal examination but only apparent on needle biopsy) [10]. He started from the basis that 0.2 cc of PCa would be clinically significant and then derived the pre-operative parameters associated with this. He determined this to be two or more positive cores with ≥ 50% core involvement of significant cancer. He set < 0.2 cc of organ-confined Gleason 3 + 3 disease as insignificant.

Winkler and colleagues in 2006 repeated the Stamey cystoprostatectomy analysis using a modern cohort and determined a clinically significant tumor volume to be ≥ 1.3 cc [11]. This is similar to the volume derived from Wolters and colleagues who analyzed the ERSPC screening database for men who had had radical prostatectomy [12].

## Can MRI help us in defining who has clinically significant PCa?

We are now in an era of MRI-informed PCa detection. Analysis of the PROMIS data suggests that MRI detects twice as many clinically significant cancers as standard TRUS biopsy and cancer lesions have different imaging characteristics to non-cancer lesions [13, 14].

We know that the use of MRI prior to a first biopsy decision will allow at least one in three to avoid an unnecessary biopsy [15]. In clinical practice, we use an MRI score of 4 or 5, or an MRI score of 3 with a high PSA density to recommend a biopsy, as we know that some men with an equivocal MRI and other risk factors such as a high PSA density will have clinically significant PCa.

There is good evidence that MRI features can help us to predict outcomes after treatment whereas a more visible disease predicts biochemical recurrence after radical treatment with surgery or radiotherapy, metastatic disease, and PCa death [16].

## Active surveillance

Active surveillance aims to defer or avoid treatment while performing serial investigations, to deliver curative treatment in a timely manner, if needed. Active surveillance is a management option for primary PCa, distinguished from watchful waiting in both scope and management. Watchful waiting is offered to men with palliative intent, in order to treat symptoms of advanced disease rather than providing a definitive cure.

The core of active surveillance is the recognition that at least half of men diagnosed with PCa on the PSA-initiated standard TRUS biopsy pathway will have cancer detected that would never become clinically apparent.

A balance exists between the survival benefits of receiving active treatment and its side effects. Active surveillance is directed at identifying the time in the natural history of PCa when the scales tip in favor of treatment. Identifying this precise time point for any given individual is one of the challenges in active surveillance. This will depend on the cancer characteristics, general health, and co-morbidities of an individual, as well as their values, preferences, and attitude to risk.

We have also seen that the likelihood of leaving active surveillance differs with the active surveillance schedule adopted. The first active surveillance protocols were aimed at men with low-risk PCa based on PSA and standard TRUS biopsy results, and standardized protocols were developed.

The canary prostate active surveillance study group has recently reported the oncological outcomes of their protocol-based active surveillance [17]. They followed 2155 patients with PSA every 3-6 months and prostate biopsy at 6*-*12 months, 2 years after diagnosis, and every 2 years thereafter. They showed that 57% of men did not undergo pathological reclassification and 53% remained free of treatment at 10 years from inclusion. The rate of metastasis or PCa-related mortality at 10 years was 1.4% and 0.1%, respectively.

The need for repeated, potentially invasive assessment, poses a significant burden to an individual patient, with studies showing anxiety rates in these men at 40% in the first year and depression rates at 20% [18]. We also know of the financial burden on healthcare systems due to the requirement for continuous follow-up and examinations. Although several mitigation strategies have been introduced in recent years, such as the use of transperineal route and rectal swabs to guide antibiotic prophylaxis, prostate biopsies continue to be an uncomfortable procedure for patients and carry a risk of sepsis and urinary retention, among other potential complications [19, 20].

A significant number of men on active surveillance choose treatment in the absence of clinical progression. This proportion was estimated at 13% within the first five years of surveillance in a Movember GAP 3 Consortium analysis [21]. Recent data has shown that the likelihood of choosing treatment in the absence of clinical progression differs with ethnicity with an analysis from the same database showing that Asian men were twice as likely to choose treatment in the absence of progression as Caucasian men (42% vs 26%) [22].

We now recognize that men choosing surveillance show a spectrum of risk, with some men very unlikely to need treatment and others where treatment can be deferred for some time, but progression is very likely to occur. There is great interest in an approach to adapt the testing during active surveillance to the risk of progression [23].

## Additional testing for men who are a low risk on the first standard biopsy

One of the challenges in the selection of patients for active surveillance is the correct identification of patients at low risk of progression.

We know that the cancers detected by different diagnostic approaches can vary widely. Barzell and colleagues reported a cohort of men with low-risk PCa on initial biopsy and were interested in active surveillance. They then underwent a combined procedure with a repeat TRUS biopsy at the same time as a transperineal multisector mapping biopsy. They demonstrated upgrading in 8–22% of patients using a repeat TRUS biopsy approach whereas a transperineal sector approach showed upgrading in 41–85% of individuals [24].

Studies on radical prostatectomy specimens of patients who are candidates for active surveillance on TRUS biopsy have shown that up to 25% harbored large anterior tumors [25, 26]. This was explained by the fact that TRUS primarily samples the posterior peripheral zone of the prostate.

MRI offers the opportunity to improve the identification of aggressive cancer. We know that MRI is better at detecting the ceiling of disease as defined by radical prostatectomy histopathology when compared to transrectal biopsy [27, 28]. One study by Dieffenbacher showed upgrading rates of 59% from standard TRUS biopsy to radical prostatectomy much higher than the 19% upgrade when MRI-targeted biopsy was used prior to radical prostatectomy [29].

The ASIST randomized study investigated MRI-targeted biopsy vs conventional biopsy in identifying men with clinically significant PCa during active surveillance [30, 31]. The addition of MRI and targeted biopsy did not show an increase in an upgrade to Gleason ≥ 3 + 4 disease at reclassification biopsy, although there was a marked center effect, where one center showed significant upgrading and two others did not. At a 2-year follow-up, the MRI arm showed a lower rate of upgrading and a 50% reduction in AS failure rate compared to the standard biopsy arm. These results demonstrate the value of MRI and targeted biopsies in better-selecting men for active surveillance, who are less likely to progress during follow-up, and in reducing failure rates of surveillance. They also raise the issue of inter-center variability with regard to MRI conduct and reporting and performing MRI-targeted biopsies. This requires following adequate standards as indicated in the prostate imaging reporting and data system (PI-RADS) and PI-QUAL recommendations, as well as developing expertise in MRI-targeted biopsies to overcome the learning curve [32–36].

## Extension of AS inclusion criteria to intermediate-risk PCa

Most of the long-term data on the safety of active surveillance derive from historical cohorts in whom risk stratification was not optimized with MRI but used TRUS biopsy and had significant heterogeneity in risk profiles. Despite this, cohorts showed very good oncological outcomes compared to initial active treatment. This was reflected in the low PCa-related mortality risk in the PROTECT trial at 15 years, where 24% and 9% of men enrolled in the monitoring arm were of D’Amico intermediate- and high-risk categories, respectively.

Data from radical prostatectomy studies have shown that rates of Gleason score downgrading for men with low percentage pattern 4 on initial biopsy were similar to rates of upgrading from Gleason 3 + 3 [37].

This supports the extension of the inclusion criteria for active surveillance in modern cohorts where MRI has become the standard of care at the time of biopsy. This has been reflected in the current PRIAS protocol. When MRI is used for risk stratification, the PRIAS inclusion criteria have now been extended to men with GS 3 + 4, PSA of ≤ 20 ng/mL, and PSA density of ≤ 0.25 ng/mL/mL. The pathological burden of disease has also been extended with no limit in Gleason 3 + 3 men and up to 50% positive cores in men with GS 3 + 4 disease.

## Risk-adapted active surveillance

The Movember International Consensus Meeting has determined a dynamic risk-stratified AS approach to be the highest-ranked research priority, where men at the lowest risk of progression would have fewer tests [38]. The traditional approaches for risk stratification use PSA, digital rectal exam, and Gleason grade. However, repeat biopsy and radical prostatectomy studies in men with low-risk cancer on TRUS biopsy, described above, show that there can be a spectrum of risk across these men, and estimating this risk can be challenging.

The Cambridge prognostic group (CPG) score comprises a 5-tier risk classification compared to the previous NICE three-tier risk stratification approach, which includes the Gleason score, PSA, and T stage [39, 40]. This five-tier approach divides Gleason 7 disease into two groups by incorporating the PSA test to distinguish a lower CPG 2 category for men with either Gleason 3 + 4 or PSA 10–20 ng/mL and a higher CPG 3 category for men with Gleason 3 + 4 and PSA 10–12 ng/mL or GS 4 + 3 disease.

## Predicting risk of progression during active surveillance

Using the traditional clinical predictive factors, some have proposed the use of machine learning to allow men at the lowest risk to have fewer tests than those who are more likely to progress [41].

Cooperberg et al have developed and validated an individualized calculator for 4-year risk prediction of progression during active surveillance [42]. This dynamic model takes into consideration seven clinical parameters including both baseline characteristics (diagnostic PSA, maximal percent positive cores, prostate size, and BMI) and subsequent PSA testing, surveillance biopsies, and time since diagnosis. The assessment of the risk of progression for men in active surveillance allows for the identification of those men at lower risk, for whom surveillance protocols may be de-intensified while maintaining close monitoring of those at higher risk. This allows fewer interventions, particularly outpatient visits and biopsies which represent the highest health-economic cost of surveillance, without missing the window of treatment of opportunity.

The Cambridge team has developed and validated a multivariable prediction model for progression to ≥ CPG3 disease at 4 years from enrollment in active surveillance [43]. This model is characterized by the addition of MRI visibility (defined as Likert or PI-RADS v 2.1 scores of 4–5) to traditionally used clinical parameters (PSA, Gleason grade, prostate volume).

## The use of MRI in risk-adapted active surveillance

Results from a Dutch cohort of Gleason 3 + 3 patients on surveillance have demonstrated that the use of MRI during follow-up can reduce the rate of unnecessary prostate biopsies [44]. They showed that avoiding biopsy in all men with PI-RADS ≤ 3 would have saved 53% (133/331) biopsies while missing reclassification in 18% (15/82) men to Gleason ≥ 3 + 4. No man with PI-RADS 3 and PSA density of < 0.15/ng/mL/mL showed progression to Gleason ≥ 3 + 4 at surveillance MRI-targeted biopsies. Similar results have been confirmed in other cohorts and been reflected in EAU guidelines, containing a recommendation that serial MRI may be adopted to avoid standard biopsy in men with very low risk of upgrading (low-risk PCa, stable MRI prostate cancer radiological estimation of change in sequential examination (PRECISE) score of 1–3, and a stable and low PSA density of < 0.15 ng/mL/cm^3^) [45, 46].

Centers are also looking at the option of using MRI as a primary monitoring tool in active surveillance [47, 48]. The UCLH cohort has demonstrated the safety and feasibility of an MRI-led active surveillance protocol where per-cause prostate biopsies are performed only in case of suspicious MRI changes or PSA fluctuations [49]. No per-protocol biopsies are performed and MRI frequency is personalized according to baseline risk and PSA kinetics. They have shown that in a population of men with GS 3 + 3 and 3 + 4 and PSA < 20 ng/mL, about 85% and 72% of men remain on MRI-led AS at 3 years and 5 years, respectively. They have described distinct event-free survival and time-to-treatment curves based on MRI visibility and Gleason grade, which may be used for risk stratification.

In order to provide a standardized framework to report serial MRIs during active surveillance, the PRECISE recommendations v.2 have been developed by an international consensus group [50]. These may be used to dynamically monitor the changes of MRI lesions during active surveillance and help guide the management and reduction of biopsies in men with stable radiological features [14]. Specifically, there is a need to define the size of a radiologically significant lesion in this context, the optimal method to determine the size of a lesion, across different characteristics of the lesion, and the optimal way to assess and report a change in size over time [50].

Some authors have suggested that biparametric MRI may be a feasible alternative to multiparametric MRI, allowing to balance the expected increased demand for scans in this population. This may be even more suitable in this lower-risk population, where avoiding false positives is a priority [51, 52]. A prospective trial is currently evaluating the additional benefit of contrast-enhanced MRI during surveillance [51]. Concerns regarding lower sensitivity for detection of progression would be mitigated by the serial monitoring of active surveillance patients, the implementation of quality control programs following PI-QUAL recommendations, and the potential introduction of artificial intelligence during active surveillance [32, 53].

Table 1 presents a summary of key studies using MRI in risk-adapted AS.

**Table 1:** Summary of key studies on MRI use in AS protocols

Study, year	Country (study period)	Cohort (*n*)	Age (yr), median (IQR)	PSA, median (IQR)	PSAD, median (IQR)	Gleason score (%)	Follow-up (mo), median (IQR)	MRI use	Biopsy use	Key results	Key messages
Schoots [44]	Netherlands (2013–2017)	331	67 (62–72)	NR	NR	GG 3 + 3 (100%)	NA	Reporting criteria: PI-RADS v2 Timing First MRI: 155/331 (47%) at diagnostic biopsy; 176/331 (53%) at confirmatory or surveillance biopsy.	Indication: men with suspicious MRI only (i.e., PI-RADS ≥ 3) Approach: MRI-targeted biopsy only	MRI results: PI-RADS 1–2: 133/331 (40%) PI-RADS ≥ 3: 198/331 (60%) Upgrading to GS ≥ 3 + 4: 198/331 (25%) -PI-RADS ≥ 3: 82/198 (41%) -PI-RADS 3: 15/50 (30%) --PSAD < 0.15 ng/mL^2^: 0/18 (0%) --PSAD ≥ 0.15 ng/mL^2^: 15/32 (47%) -Pi-RADS 4: 36/101 (36%) -PI-RADS 5: 31/47 (66%) Stratifying men upgraded to GS ≥ 3 + 4 and PI-RADS 3 (15/50, 30%) by PSA Density: --PSAD < 0.15 ng/mL^2^: 0/18 (0%) --PSAD ≥ 0.15 ng/mL^2^: 15/32 (47%) Upgrading to GS ≥ 4 + 3: 24/331 (7%) -PI-RADS ≥ 3: 82/198 (41%) - PI-RADS 3: 4/50 (8%) -Pi-RADS 4: 8/101 (8%) -PI-RADS 5: 12/47 (26%) Upgrading to GS ≥ 8: 11/313 (3%).	Stratification with a combination of PI-RADS and PSA-density may reduce unnecessary additional MRI biopsy testing. No men with PI-RADS 3 and PSA density of < 0.15 ng/mL/mL showed progression to Gleason ≥ 3 + 4 at surveillance MRI-targeted biopsies. Avoiding biopsy in all men with PI-RADS ≤ 3 would have saved (133/331) biopsies while missing reclassification in 18% (15/82) men to Gleason ≥ 3 + 4.
Bhanji [45]	United States (2016–2020)	552	65 (60–69)	5.3 (4.3–7.0)	0.11 (0.08–0.16)	GS 3 + 3 (100%)	NA	Reporting criteria: PI-RADS Timing 107 (only pre-diagnostic biopsy only); 350 (at pre-confirmatory biopsy only); 65 (both at diagnostic and confirmatory biopsy).	Indication: Confirmatory biopsy Approach: MRI-targeted + systematic	Reclassification to GG 2: 103/522 (20%) -MRI positive (PI-RADS ≥ 3): 83/306 (27%) -MRI negative (PI-RADS ≤ 2): 20/218 (9.2%) Reclassification to NCCN unfavorable intermediate risk PCa: 61/522 (12%) -MRI positive (PI-RADS ≥ 3): 49/306 (16%) -MRI negative (PI-RADS ≤ 2): 12/218 (5.5%) Reclassification to GG ≥ 3: 29/522 (5.6%) -MRI positive (PI-RADS ≥ 3): 24/306 (7.8%) -MRI negatived (PI-RADS ≤ 2): 5/218 (2.3%)	Reclassification rates at CBx were high for men with positive MRI but < 10% for any reclassification event in men with negative MRI. Our data support CBx (systematic D targeted) for men with positive MRI considering AS, whereas men with GG1 cancer and negative MRI may be able to avoid CBx until a routine surveillance biopsy in 2–3 years.
Stavrinides [49]	UK (2004–2017)	672	62 (56–66)	6 (4.5–8.4)	0.12 (0.09–0.18)	GS 3 + 3: 524 (78%) GS 3 + 4: 148 (22%)	58 mo (37–82)	Reporting criteria: Likert 1–5; PRECISE 1–5 Timing: At baseline; 12 months; at 24 months if visible disease; PSA-based MRI thereafter.	Indication: Surveillance biopsy upon MRI, clinical, or PSA changes. Technique: Transperineal, MRI-targeted	Events: -Treatment: 216/672 (32.1%; 62.0 patients/1000 py) -Transition to watchful waiting: 21(672 -Metastases: 8/672 (2.1 events /1000 py) -Pca-related deaths: 0% -Non-PCa-related deaths: 24/672 (6.3 events /1000 py) -Upgrade to GS ≥ 4 + 3: 27/672 (7.7 events/1000 py) Event-free survival: -3 years: 84.7% -5 years: 71.8% 3-year vent-free survival -GS 3 + 3 Nonvisible: 83.4% -GS 3 + 3 MRI visible: 72.3% -GS 3 + 4 Nonvisible: 62.8% -GS 3 + 4 MRI visible: 33.8%	The rates of discontinuation, mortality, and metastasis in MRI-led surveillance are comparable with those of standard AS. MRI-visible disease and/or secondary Gleason grade 4 at baseline are associated with a greater likelihood of moving to active treatment at 5 years.
Giganti [14]	UK (2005–2020)	553	62 (56–67)	6.3 (4.7–8.4)	0.12 (0.09–0.2)	GS 3 + 3: 445 (80%)GS 3 + 4: 108 (20%)	74.5 mo (53–98)	Reporting criteria: Likert 1–5; PRECISE 1–5 Timing: At baseline; further MRI depending on baseline risk and clinical changes	Indication: Surveillance biopsy upon MRI, clinical, or PSA changes. Technique: Transperineal, MRI-targeted	Baseline PI-RADS score: -1–2: 266 (48%) -3: 104 (19%) -4–5:183 (33%) Overall PRECISE score: -1–2: 123/553 (22%) -3 (non visible): 152 /553 (28%) -3 (visible): 38/553 (7%) -4–5: 240/553 (43%) Clinical progression (upgrade to GS ≥ 4 + 3 or treatment): 165/553 (30%) -PRECISE 1–3: 17/313 (5%) -PRECISE 4–5: 146/240 (61%)	Patients without radiological progression on MRI (PRECISE 1–3) during AS had a very low likelihood of clinical progression and many could avoid routine re-biopsy. Clinical progression was almost always detectable in patients with radiological progression on MRI (PRECISE 4–5) during AS. Patients with radiological progression on MRI (PRECISE 4–5) during AS showed a trend of an increase in PSA density.
Thankapannair [54]	England, UK (2019–2022)	156	67.3 (62–71)	6.1 (4.5–7.9)	0.12 (0.08–0.17)	GS 3 + 3: 113 (72%) GS 3 + 4: 43 (28%)	19 (13–22)	Pre-biopsy MRI F-U strategy: based on STRATCANS group	**Indication:** Per protocol biopsy based on STRATCANS group; per cause biopsy if any change.	Progression to CPG3: -STRATCANS 1: 0 -STRATCANS 2: 2/61 (3.3%) -MRI non visible: 0 -MRI non-visible: 2/41 (4.9%) -STRATCANS 3: 2/29 (7.4%)	STRATCANS offers a risk-stratified follow-up schema that may de-escalate follow-up in men at lower risk of progression during AS and reduce resource utilization.

*AS* active surveillance, *GS* Gleason score, *PSA* prostate-specific antigen (ng/mL), *PSAD* PSA density (ng/mL/mL), *STRATCANS* stratified cancer surveillance

## The development of MRI-enhanced active surveillance schedules

Based on the development of the Cambridge Prognostic grouping, the same authors have developed STRATCANS, a risk stratification approach to active surveillance using the CPG in combination with PSA density [54] (Table 2). They have described three groups: STRATCAN 1 (CPG 1 and PSA density < 0.15 ng/mL); STRATCAN 2 (CPG2 or PSA density ≥ 0.15 ng/mL); STRATCAN 3 (CPG2 and PSA density ≥ 0.15 ng/mL). They have applied a stratified follow-up schedule, where all patients have a three-monthly PSA, but the use of outpatient telephone calls biopsy, and MRI differs within each group. In STRATCANS groups 1 and 2, the timing of the first repeat MRI during follow-up is further stratified into three follow-up schedules based on the baseline MRI Likert score: at 3 years (Likert 1–2), at 18 months (Likert 3) and at 1 year (Likert 4–5); in STRATCANS 3, all patients have a repeat MRI at 1 year.

**Table 2:** STRATCANS risk stratification and study results

CAMBRIDGE STRATCANS			STRATCANS 1	STRATCANS 2	STRATCANS 3
Inclusion criteria CPG 1 = GS 3 + 3, PSA < 10 ng/mL, ≤ cT2. CPG 2 = GS 3 + 3, PSA 10–20, ≤cT2; OR GS 3 + 4, PSA < 10, ≤ cT2			CPG 1 AND PSAD < 0.15, cT ≤ 2	CPG 2 OR PSAD ≥ 0.15, cT ≤ 2	CPG 2 AND PSAD ≥ 0.15, cT ≤ 2
Follow-up schedule	PSA		3 monthly		
	Telephone consultation		18 monthly	12 monthly	6 monthly
	Baseline MRI Likert	1–2	Repeat at 3 years		Repeat at 12 months (any Likert)
		3	Repeat at 18 months		
		4–5	Repeat at 12 months		
	Re-biopsy		No routine re-biopsy. Triggered if any change.	Re-biopsy at 3 years. Triggered if any change.	No routine re-biopsy. Triggered if any change.
Study population					
STRATCANS category	All		STRATCANS 1	STRATCANS 2	STRATCANS 3
Enrolled	156		66/156 (42.3%)	61/156 (39.1%)	29/156 (18.6%)
Visible lesion			35/65 (53.8%)	19/61 (31.1%)	Not reported
Study outcome					
Still on AS	133/156 (85.3%)		61/66 (95.3%)	53/61 (91.3%)	19/29 (70.3%)
Exited AS	21/156 (13.5%)		5/66 (7.6%)	8/61 (13.1%)	10/29 (34.5%)
Progression to CPG ≥ 3 (based on pathological progression)	4/156 (2.6%)		0	2/61 (3.3%)	2/29 (6.9%)
Metastasis	0%		0%	0%	0%

*AS* active surveillance, *CPG* Cambridge Prognostic Group, *GS* Gleason score, *PSA* prostate-specific antigen, *STRATCANS* stratified cancer surveillance

They presented the results of the application of STRATCANS to an ongoing cohort of active surveillance patients and further stratified outcomes based on baseline MRI visibility (Likert 3–5 vs 1–2). They reported no progression to CPG ≥ 3 in both STRATCANS 1 and the STRATCANS 2 subgroup with non-MRI visible PCa. Of men in the highest risk group, characterized by Gleason 3 + 4 and the PSA of 10–20 ng/mL, they report a low rate of progression with two in 29 men progressing to CPG 3, of which only one to Gleason ≥ 4 + 3.

It is notable that in the cohort of 156 men, 66 were deemed to be in STRATCANS 1 with no MRI visible disease and a PSA density < 0.15 ng/mL. In a modern MRI-led approach, these men would not have undergone a biopsy, and it is therefore not surprising that they did not progress during two years of active surveillance.

## Conclusions

The heterogeneity of men enrolled in active surveillance mandates a risk-stratified approach, to diminish the psychological and economic burden of surveillance while ensuring timely detection of progression to guarantee treatment. Traditional standardized approaches to surveillance have benefited from the introduction of MRI in practice as an instrument to improve baseline risk stratification and reduce active surveillance failure rates. Current active surveillance cohorts have begun to use MRI to further guide follow-up and postpone protocol biopsies or replace them altogether.

Further studies are necessary to define and validate the use of risk-stratified active surveillance protocols where MRI can be appropriately adopted for baseline stratification and dynamic monitoring.

## Abbreviations

MRI: Magnetic resonance imaging
PCa: Prostate cancer
PI-RADS: Prostate imaging reporting and data system
PRECISE: Prostate cancer radiological estimation of change in sequential examination
PSA: Prostate-specific antigen
TRUS: Transrectal ultrasound
